# MiRNA-Embedded ShRNAs for Radiation-Inducible LGMN Knockdown and the Antitumor Effects on Breast Cancer

**DOI:** 10.1371/journal.pone.0163446

**Published:** 2016-09-22

**Authors:** Zhi-Qiang Zhang, Zhi Cao, Cong Liu, Rong Li, Wei-Dong Wang, Xing-Yong Wang

**Affiliations:** 1 Ministry of Education Key Laboratory of Child Development and Disorders, Chongqing, 400014, China; 2 Key Laboratory of Pediatrics in Chongqing, Chongqing, 400014, China; 3 Chongqing International Science and Technology Cooperation Center for Child Development and Disorders, Chongqing, 400014, China; 4 Department of Critical Care Medicine, Children's Hospital of Chongqing Medical University, Chongqing, 400014, China; 5 Institute of Combined Injury, State Key Laboratory of Trauma, Burns and Combined Injury, College of Preventive Medicine, Third Military Medical University, Chongqing, 400038, China; 6 Department of Radiation Oncology, Sichuan Cancer Hospital, Chengdu, 610041, China; University of South Alabama Mitchell Cancer Institute, UNITED STATES

## Abstract

Legumain (LGMN) is highly expressed in breast cancer (BC) and other solid tumors and is a potential anticancer target. Here we investigate the anti-tumor effects of short hairpin RNAs (shRNAs) targeting LGMN embedded in a microRNA-155 (miR-155) architecture, which is driven by a radiation-inducible chimeric RNA polymerase II (Pol II) promoter. Lentiviral vectors were generated with the chimeric promoter which controlled the expression of downstream shRNA-miR-155 cassette. Fluorescence was observed by using confocal microscopy. Real-time quantitative PCR and Western blotting were used to determine the expression level of LGMN, MMP2, and MMP9. Furthermore, the proliferation and invasive ability of BC cells was analyzed via plate colony formation and invasion assays. Here we demonstrated that the chimeric promoter could be effectively induced by radiation treatment. Furthermore, the shRNA-miR-155 cassette targeting LGMN could be effectively activated by the chimeric promoter. Radiation plus knockdown of LGMN impairs colony formation and dampens cell migration and invasion in BC cells. Inhibition of LGMN downregulates MMP2 and MMP9 expression in BC cells. Pol II-driven shRNA-miR-155 could effectively suppress the growth and invasiveness of BC cells, and that the interference effects could be regulated by radiation doses. Moreover, knockdown of LGMN alleviates the aggressive phenotype of BC cells through modulating MMPs expression.

## Introduction

BC is one of the most common malignant tumors in females which severely affects the mental and physical health of women [1, 2]. Tumor metastasis is one of the causes of BC mortality and has important functions in the diagnosis and treatment of BC. The invasiveness of tumors and the microenvironment surrounding tumors are important influencing factors for the occurrence of tumor metastasis [3]. LGMN is a cysteine endopeptidase that is localized in lysosomes. It is highly expressed not only in a variety of solid tumor cells but also in tumor neovascular endothelial cells and tumor-associated macrophages [4]. However, it is not expressed or has low expression status in normal tissues [5]. Studies have shown that LGMN not only participates in tumor migration and invasion but also plays important roles in the occurrence and development of tumors; furthermore, it is associated with a poor prognosis in tumor patients [5–7]. Therefore, the targeted silencing of LGMN expression in tumor cells is a valuable treatment method.

RNA interference (RNAi) can be used to specifically bind cellular mRNA to homologous sequences via double-stranded RNA and to degrade the mRNA of target genes, thus achieving specific inhibition of target gene expression, which plays an important role in gene therapy for tumors. Currently, commonly used small interfering RNA (siRNA) can only generate transient gene inhibition effects and short-term interference effects; therefore, its clinical application is limited. Promoters used in shRNA interference plasmids mainly consist of Pol III promoters, which do not have tissue specificity and can be persistently expressed in almost all cells. Therefore, in addition to killing tumor cells during treatment, these promoters can also damage normal tissues [8, 9], thus restricting their clinical application. In recent years, plasmids with shRNA embedded in microRNA (miRNA) backbone driven by Pol II promoters were used in RNAi [9–11], such “shRNAmir” structures serve as natural substrates in miRNA biogenesis pathways and can strongly increases mature shRNA levels and knockdown efficacy [12, 13]. Using this method, in addition to optimizing the interference effects and reducing off-target effects, the tissue specificity of this group of plasmids can be developed, and RNAi can be regulated using the temporal and spatial specificity method, therefore improving the targeting and safety of gene therapy [14, 15].

The expression level of the Egr-1 gene is increased in cells due to stress after radiation. Six CC(A+T)_6_GG regulatory elements (CArG elements) are present upstream of its 5’ end. The element can sense radiation stimulation to drive transcription and is the direct site of action of oxygen free radicals [16]. Studies including our research group and other labs have confirmed that the chimeric promoter constructed by tandem series of CArG regulatory elements coupled with the Pol II basic promoter could significantly enhance the expression level of downstream genes[17–19]. This chimeric promoter could control the downstream genes expressing within a limited time during the radiation period, also strictly limited to the range of the radiation field.

In this study, we used nine tandem CArG elements coupling with CMV basic promoter to construct the chimeric promoter C_9_BC, and then constructed shRNAmir-based lentiviral plasmids targeting LGMN, which is driven by the C_9_BC Pol II promoter. After lentiviral packaging, viruses were used to infect SK-BR-3 and MDA-MB-231 cells to establish stably transfected cells. The results showed that the C_9_BC promoter exhibited a significant radiation-inducible ability after radiation treatment. Furthermore, radiation plus knocking-down of LGMN could effectively inhibit the growth and invasiveness of BC cells.

## Materials and Methods

### Cell lines

293FT cells were purchased from Invitrogen (Carlsbad, California, USA). The SK-BR-3 and MDA-MB-231 cell line were obtained from the American Type Culture Collection (ATCC, Manassas, VA, USA). Both cells were cultured in DMEM high glucose culture medium containing 10% fetal bovine serum at 37°C in a 5% CO_2_ incubator.

### Confocal microscopy

Cells were plated onto MatTek glass-bottomed culture dishes and allowed to adhere overnight. After treatments, cells were washed with PBS and fixed with 4% paraformaldehyde. Images were recorded using a Zeiss confocal microscope.

### Real-time reverse transcription quantitative PCR

Total RNA was extracted by following the RNAiso Plus protocol (TaKaRa). Reverse transcription was performed according to the PrimeScript RT reagent Kit protocol (TaKaRa). Real-time PCR was performed using SYBR Premix Ex Taq (TaKaRa). The internal control was GADPH. The primers used were shown in Table A in S1 File. The quantitative PCR reaction used the two-step method (Bio-Rad Q5). For quantitative analysis, all samples were analyzed using the ΔΔCT value method.

### Western blotting

The cells were dissociated, centrifuged, and collected. Cell lysis buffer (Beyotime company, Jiangsu, China) was added, and the cells were incubated in an ice bath for 15–20 min. The cells were centrifuged at 4°C at 20000 rpm for 10 min. The supernatant was collected for future use. Proteins were quantified using the BCA method (Beyotime), and the concentrations of samples in all groups were adjusted to the same level. Loading buffer was added to the samples; after boiling in water for 5 min, the samples were stored at -20°C for subsequent use. A 12% SDS-polyacrylamide gel was prepared, and the samples were subjected to electrophoresis. The samples were then transferred onto a 0.2 μm polyvinylidene fluoride (PVDF) membrane using the wet transfer method at 90 V. After membrane transfer was complete, the PVDF membrane was removed and blocked for 1 h at room temperature. Primary antibodies were added and incubated at 4°C overnight. After the membrane was washed with phosphate-buffered saline/tween (PBST), a horseradish peroxidase-labeled secondary antibody was added, and the membrane was incubated at room temperature for 1 h. After the membrane was washed with PBST, the proteins were detected using the enhanced chemiluminescence (ECL) method (Byotime). β-actin was used as a control and obtained from Beyotime Biotechnology. All of the primary antibodies and dilutions contained the following: goat anti-LGMN antibody (1:500; R&D, Shanghai, China), mouse anti-MMP2 antibody (1:1000; ZSGN-BIO, Beijing, China), and mouse anti-MMP9 antibody (1:1000; ZSGN-BIO). Secondary antibodies coupled to horseradish peroxidase (HRP) were purchased from Beyotime.

### Plate colony formation experiments

Cells in the logarithmic growth phase were dissociated and centrifuged. The cell pellet was resuspended, and the cell concentration was adjusted to 1×10^5^/mL. Cell suspensions of 100 μL were inoculated onto 6-cm plates; after culture medium was added, the cells were placed in an incubator for conventional culturing for 2–3 weeks. When visible colonies appeared, the culture was terminated. The cells were washed with PBS twice, fixed in methanol for 15 min, stained with crystal violet for 20 min, washed with tap water, and air dried. The number of visible colonies was counted using a transparent film with grids. Colony formation rate = (number of colonies/number of inoculated cells) ×100%.

### Invasion experiments

Matrigel (Invitrogen) was transferred from -20°C to 4°C for 12 h to obtain a liquid state for subsequent use. The upper chamber surface of the basement membrane of the transwell (Corning, NY, USA) was coated with a 50 mg/L matrigel 1:8 diluted solution and air dried at room temperature. Then, 50 μL of serum-free culture medium containing 10 g/L BSA (Gibco, Grand Island, NY, USA) was added to each well. The basement membrane was hydrolyzed at 37°C for 30 min. Cells were starved by serum withdrawal for 12–24 h. The cells were collected using routine dissociation, centrifuged, and resuspended in serum-free medium. The cell density was adjusted to 1×10^5^/ml, and 200 μL was added to the transwell. The bottom chamber was filled with 500 μL of culture medium containing 20% BSA. After conventional culturing for 24 h, the transwell was removed, and the cells were eluted with PBS. Cells on the inner membrane were wiped using a cotton swap, fixed in 95% ethanol for 5 min, stained with crystal violet, and counted.

### Scratch Wound Healing Assay

The cells were inoculated onto six-well plates and cultured at 37°C in a 5% CO_2_ cell incubator. After the cells reached 70%-80% confluence, cross lines were made using a 200-μL sterile pipette tip. The cells were washed three times with sterile PBS to remove the scratched cells. The cells were continuously cultured in serum-free culture medium. After 0 h, 24 h, and 48 h, the cells were photographed. Cell migration distance = distance at 0 h-distance at 48 h.

### Statistical methods

All statistical analyses were performed with SPSS for Windows version 13.0 (SPSS Inc., Chicago, IL, USA). The data are presented as the mean±standard deviation (SD). Error bars represent the SD of three independent experiments. Differences between groups were compared using ANOVA. All statistical tests were two-sided, and differences were considered statistically significant when P<0.05.

## Results

### The radiation-induced expression features of C_9_BC chimeric promoter

**Fig 1:**
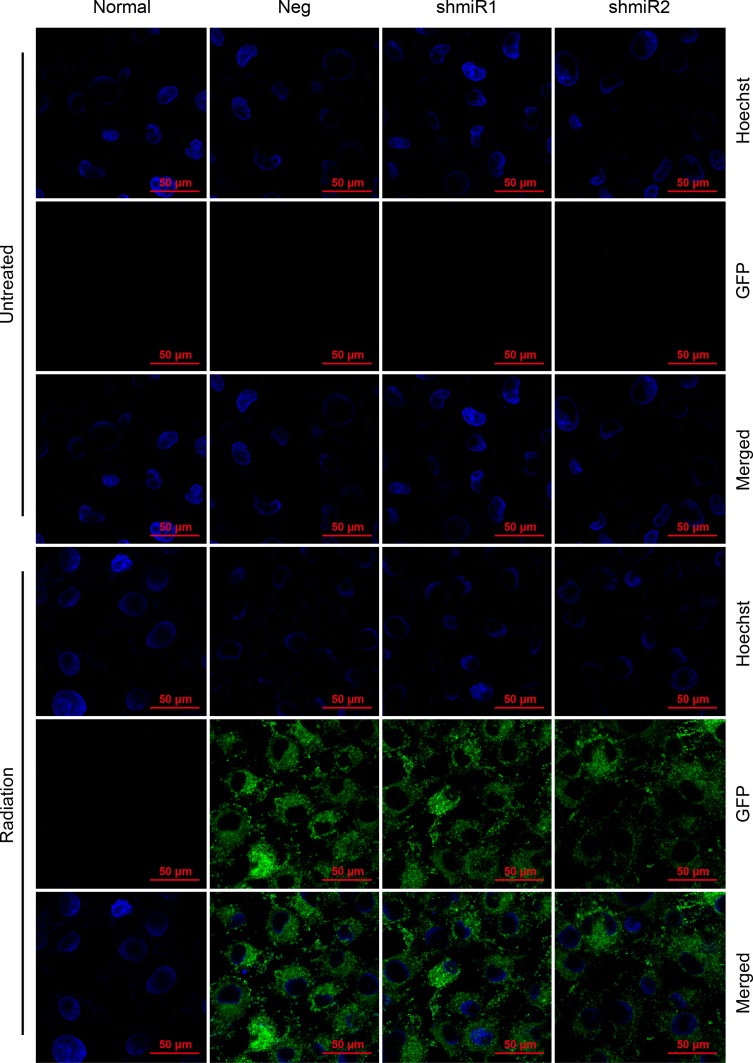
GFP expression in SK-BR-3 cells treated for 48h by laser confocal microscopy. The figures showed above were merged photos in which cell nucleus was dyed with Hoechst 33342. The green fluorescence was prominent after 48 hours. Normal: cells without treatment; Neg: cells stably expressed shRNA-neg-miR-155; shmiR1: cells stably expressed shRNA-1-miR-155; shmiR2: cells stably expressed shRNA-2-miR-155; Radiation: cells receiving 6 Gy of radiation treatment.

Our previous experimental results showed that the chimeric promoters containing CArG elements could be effectively activated in a radiation dosage of 6Gy[17]. Accordingly, 6 Gy was used as the radiation dose in this study. S1 Fig shows that after the stably transfected cell line SK-BR-3 received 6 Gy of radiation treatment, significant green fluorescence protein (GFP) expression was observed by using laser confocal microscopy; this expression was even more evident after 48 h (Fig 1). The above results indicated that the C_9_BC chimeric promoter could effectively drive downstream gene transcription after radiation treatment.

### Radiation exposure resulted in knockdown of LGMN

**Fig 2:**
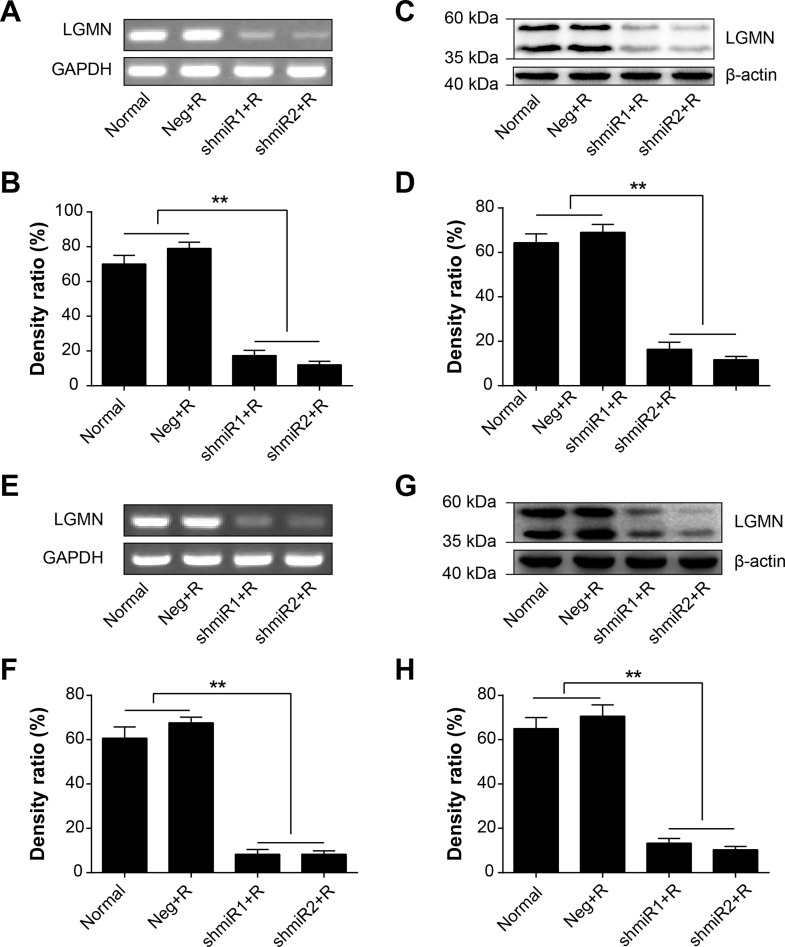
Radiation exposure initiates knockdown of LGMN. (**A)** LGMN mRNA in SK-BR-3 cells was detected by RT-PCR after 24 hs of radiation exposure. GAPDH served as an internal control. **(B)** Quantification of LGMN mRNA expression in SK-BR-3 cells by RT-PCR. **(C)** LGMN protein in SK-BR-3 cells was detected by Western blotting after 72 hs of radiation exposure, β-actin served as an internal control. **(D)** Quantification of LGMN protein expression in SK-BR-3 cells by Western blotting. (**E)** LGMN mRNA in MDA-MB-231 cells was detected by RT-PCR after 24 hs of radiation exposure. **(F)** Quantification of LGMN mRNA expression in MDA-MB-231 cells by RT-PCR. **(G)** LGMN protein in MDA-MB-231 cells was detected by Western blotting after 72 hs of radiation exposure. **(H)** Quantification of LGMN protein expression in MDA-MB-231 cells by Western blotting. **P<0.01. Data was shown as the means ± SD from three independent experiments. The group definition is the same as Fig 1.

The C_9_BC chimeric promoter could effectively activated downstream GFP expression after 6 Gy of radiation treatment. To determine whether the shRNA-miR-155 cassette targeting LGMN could also be effectively activated by the C_9_BC chimeric promoter, we measured LGMN expression from both mRNA and protein levels. Since the C_9_BC chimeric promoter can be activated after 24 hours of radiation treatment, so we collected cells at 24 h after radiation treatment to extract mRNA for RT-PCR and quantitative PCR analysis (Fig 2A, 2B, 2E and 2F), and we collected cells at 72 h to extract proteins for Western blot analysis (Fig 2C, 2D, 2G and 2H). As demonstrated in Fig 2A–2H, after exposure to 6 Gy of radiation, the mRNA and protein expression levels of LGMN were both significantly decreased compared to those of the other groups.

### Radiation-induced knockdown of LGMN combined with radiation impairs colony formation

**Fig 3:**
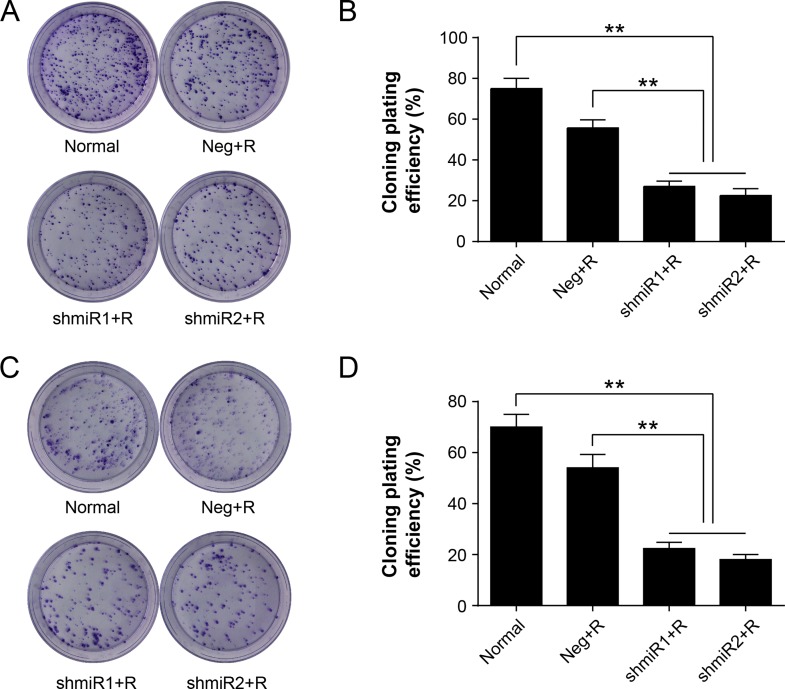
Radiation induced knockdown of LGMN impairs colony formation. **(A)** Representative images of clone-forming assay of SK-BR-3 cells receiving different treatments. **(B)** Comparison of cloning efficiency of SK-BR-3 cells. **(C)** Representative images of clone-forming assay of MDA-MB-231 cells receiving different treatments. **(D)** Comparisons of cloning efficiency of MDA-MB-231 cells. ** *P*<0.01. Data was shown as the means ± SD from three independent experiments. The group definition is the same as Fig 1.

It is reported that increased expression of LGMN might affect cell proliferation and be involved in liver carcinogenesis[20]. To ascertain whether or not expression levels of LGMN affects cell proliferation and/or synergizes with radiation treatment, we further compared colony formation rates among different treatment groups in SK-BR-3 and MDA-MB-231 cell lines. The results of the plate colony formation experiments in each treatment groups are shown in Fig 3. Differences between different treatment groups were compared by calculating the colony formation rates. The experimental results showed that the colony formation ability of the shmiR1+R or shmiR2+R group was significantly suppressed compared with other groups in both cell lines. Furthermore, the colony formation rate was obviously reduced in the Neg+R group in both cell lines. There was no significant difference between shmiR1+R and shmiR2+R group in colony formation rates.

### Knockdown of LGMN combined with radiation suppresses cell migration and invasion

**Fig 4:**
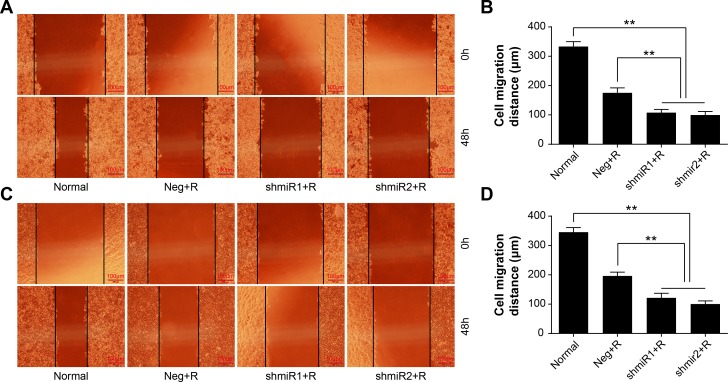
Scratch wound healing assay in different treatment groups. (**A)** Representative images of the wound healing assay in SK-BR-3 cells. (**B)** Comparisons of migration distances in SK-BR-3 cells. **(C)** Representative images of the wound healing assay in MDA-MB-231 cells. **(D)** Comparisons of migration distances in MDA-MB-231 cells. ** *P*<0.01. Data was shown as the means ± SD from three independent experiments. The group definition is the same as Fig 1.

**Fig 5:**
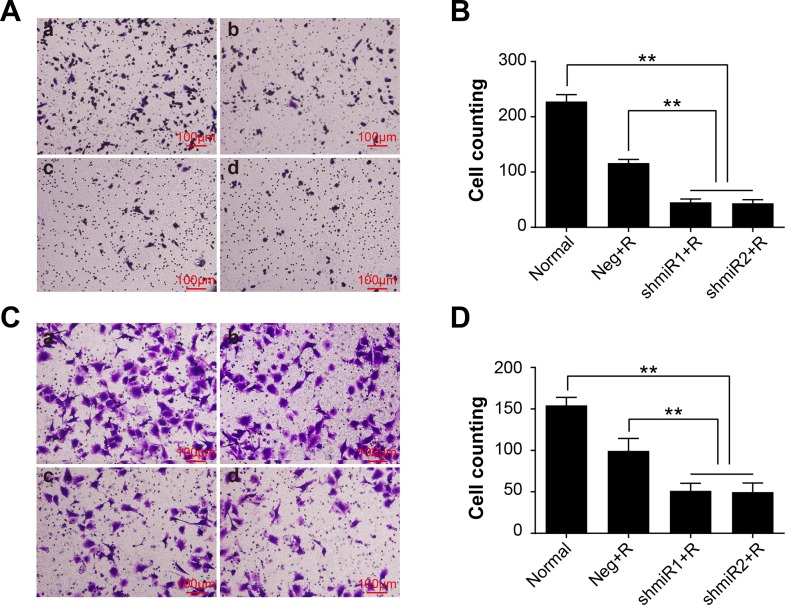
Transwell invasion assay in different treatment groups. **(A)** Representative images of the invasion assay in SK-BR-3 cells. (a) Normal; (b) Neg+R; (c) shmiR1+R; (d) shmiR2+R. **(B)** Comparisons of the cell counts in different groups of SK-BR-3 cells. **(C)** Representative images of the cell invasion assays in MDA-MB-231 cells. (a) Normal; (b) Neg+R; (c) shmiR1+R; (d) shmiR2+R. **(D)** Comparisons of the cell counts in different groups of MDA-MB-231 cells. ** *P*<0.01. Data was shown as the means ± SD from three independent experiments. The group definition is the same as Fig 1.

It is reported that high LGMN expression could facilitate metastasis and invasion of tumor cell. To verify weather LGMN knockdown plus radiation treatment could effectively inhibit metastasis and invasion features of BC, we calculated the migration and invasion abilities of SK-BR-3 and MDA-MB-231 cells in different treatment groups. The experimental results in Fig 4 and Fig 5 indicate that after radiation treatment, the migration and invasion abilities of LGMN knockdown cells of the two cell lines were significantly suppressed compared to those in the other groups; the differences were statistically significant (*P*<0.01). Additionally, in both cell lines, the migration and invasion abilities were also suppressed in the Neg+R groups compared with the Normal groups. There were no significant differences between shmiR1+R and shmiR2+R groups in cell migration and invasion features in these two cell lines (*P*>0.05).

### Knockdown of LGMN downregulates MMP2 and MMP9 expression

**Fig 6:**
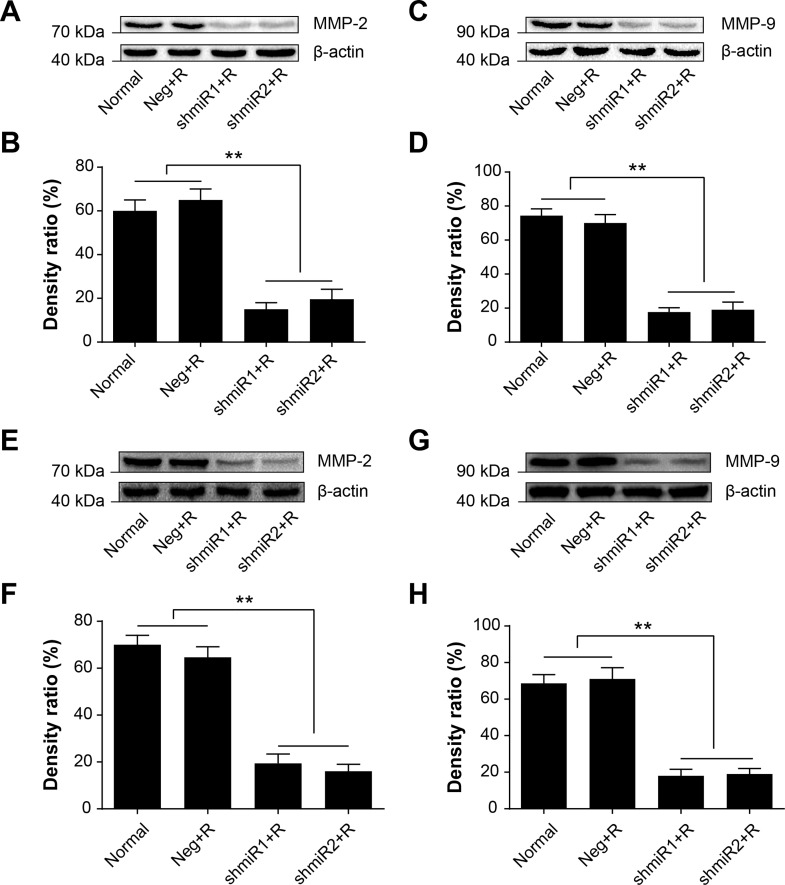
Radiation-induced knockdown of LGMN downregulates MMP2 and MMP9 expression. **(A,C)** MMP2 or MMP9 protein in SK-BR-3 cells was detected by Western blot after 72 hs of radiation exposure, β-Actin served as an internal control. **(B,D)** Quantification of MMP2 or MMP9 protein expression in SK-BR-3 cells. **(E,G)** MMP2 or MMP9 protein in MDA-MB-231 cells was detected by Western blot after 72 hs of radiation exposure, β-Actin served as an internal control. **(F,H)** Quantification of MMP2 or MMP9 protein expression in MDA-MB-231 cells. ** *P*<0.01. Data was shown as the means ± SD from three independent experiments. The group definition is the same as Fig 1.

Data presented above indicated that the radiation-induced knockdown of LGMN could suppress migration and invasion features of BC cells. It has been reported that degradation of the extracellular matrix is an essential step in the formation of tumor metastasis. The mechanism of radiation-induced knockdown of LGMN suppressing cell migration and invasion was next investigated with a focus on MMP9 and MMP2, a kind of gelatinase has the unique ability to degrade extracellular matrix components and facilitate tumor migration and invasion. Fig 6 showed that radiation-induced knockdown of LGMN in shmiR1+R and shmiR2+R groups resulted in a significant reduction of MMP2 and MMP9 protein levels. There was no significant differences between shmiR1+R and shmiR2+R group in MMP2 and MMP9 protein levels in both cell lines (*P*>0.05).

## Discussion

LGMN mainly localizes in lysosomes. Its gene sequence is highly conserved, and it is a member of the cysteine peptidases C13 family. It is not expressed or exhibits low expression levels in normal tissues; however, it is highly expressed in many types of solid tumors including cervical cancer, stomach cancer, colon cancer, ovarian cancer, and BC [21–24]. Many studies have indicated that LGMN affects tumor invasion and metastasis and is closely associated with a poor prognosis [25]. Studies have also shown that LGMN plays a role in the proliferation and apoptosis process of tumors [20, 25]. Because LGMN is closely associated with tumor invasion, metastasis, and the microenvironment variations, using LGMN as a target for gene therapy could have an important value for clinical application. So far, there is no study that reported the knockdown of LGMN combined with radiation treatment. In this study, we used LGMN as a therapeutic target to observe the anti-tumor effects of radiation-induced LGMN knockdown in BC cells. We showed that the shRNAmir structure targeting LGMN plus radiation treatment could significantly reduce the expression of LGMN in mRNA and protein levels, decrease the proliferation ability, and inhibit the migration and invasiveness of BC cells.

RNAi is a biological process of inhibiting gene expression by RNA molecules through the degradation of specific mRNA molecules. siRNA is a commonly used and effective method of RNAi. It acts rapidly but can only exert transient gene inhibition effects; in addition, it cannot be amplified. Therefore, its systemic application at the *in vivo* level is limited. Commonly used shRNA interference plasmids can maintain long-term target gene inhibition effects, however, the promoters in shRNA plasmids mainly belong to the Pol III promoters, which lacks targeting features and expresses in almost all cells. Therefore, their application for treatment at the *in vivo* level is limited. Studies have shown that adding miRNA arms at both ends of shRNA and coupling this cassette with Pol II promoters to simulate endogenous knockdown of mRNA by miRNA produces the advantages of high knockdown efficiency and low off-target effects [26, 27]. To address above questions, we constructed a chimeric radiation-inducible Pol II promoters which coupling with shRNAmir structure to limit the targeted gene knockdown strictly in cancer cells.

Utilization of radiation induction to control gene therapy should not only allows real-time regulation of expression levels of target genes but also achieves tumor-targeting treatment effects and a synergistic effect between radiotherapy and gene therapy at the same time. The CArG elements in the Egr-1 gene can effectively activate downstream gene transcription under radiation induction and temporally and spatially perform targeted regulation of gene expression[28, 29]. Our previous studies have shown that promoters containing tandem CArG elements could be induced by radiation treatment and drive the expression of the downstream therapeutic genes [17, 30]. Therefore, this chimeric element can be used as a radiation-sensitive regulatory element to control the expression of therapeutic genes. Our studies have shown that the C_9_BC chimeric promoter produces better radiation induction characteristics and lower background expression levels than the Egr-1 promoter. This chimeric promoter belongs to the Pol II promoters, therefore, the shRNAmir structure can be linked to its downstream so as to control gene knockdown by radiation treatment. Our study showed that the C_9_BC chimeric promoter could effectively activate downstream gene expression after exposure to 6 Gy of radiation and produce an RNAi effect for targeting LGMN to achieve an effective anti-tumor activity. Furthermore, we found radiotherapy and targeted LGMN knockdown had synergistic effects in BC cells, so, knockdown of LGMN plus radiation treatment could enhance the therapeutic effects so as to gain more positive anti-tumor effects.

It has been reported that cancer cells overexpressing LGMN possessed increased migratory and invasive activity *in vitro*[31, 32]. DNA vaccine targeting LGMN could suppress angiogenesis, cell growth, and metastasis in murine tumor models[33]. Consistent with previous findings, we found that LGMN play an important role in cell migration and invasion in BC cells. To elucidate the mechanism involved in BC cell invasion and metastasis, we further evaluated the metalloendopeptidases MMP2 and 9 expressions in different cell samples. We found that knockdown of LGMN could downregulate the expression of MMP2 and MMP9, which could break down extracellular matrix and promote tumor metastasis. Therefore, knockdown of LGMN should alleviates the aggressive phenotype through downregulate MMPs in BC cells.

In summary, for the first time, we utilized C_9_BC as a radiation-inducible promoter to drive the expression of shRNA-miR-155 cassette targeting LGMN in the study of BC cells. The combination of radiation treatment and LGMN gene knockdown could effectively suppress cell growth and invasiveness of BC cells. Furthermore, the effective temporal and spatial control of RNAi should increase the accuracy and safety of anti-tumor treatment.

## Supporting Information

S1 Fig GFP expression in SK-BR-3 cells treated for 24h by laser confocal microscopy. (TIF)

S1 File Primers used in this study (Table A). GFP expression in SK-BR-3 cells treated for 24h by laser confocal microscopy (S1 Fig caption). Construction of shmiR plasmids with miR-155 flanking sequences (Protocol A). Construction of lentiviral plasmids containing radiation-inducible promoters and shRNA-miR-155 cassette (Protocol B). Lentivirus packaging and infection (Protocol C).
(DOCX)
