# Supplementary figures and images for: MiRNA-Embedded ShRNAs for Radiation-Inducible LGMN Knockdown and the Antitumor Effects on Breast Cancer
Source: PLoS One. 2016 Sep 22;11(9):e0163446. doi: 10.1371/journal.pone.0163446 (PMC5033420; doi:10.1371/journal.pone.0163446)

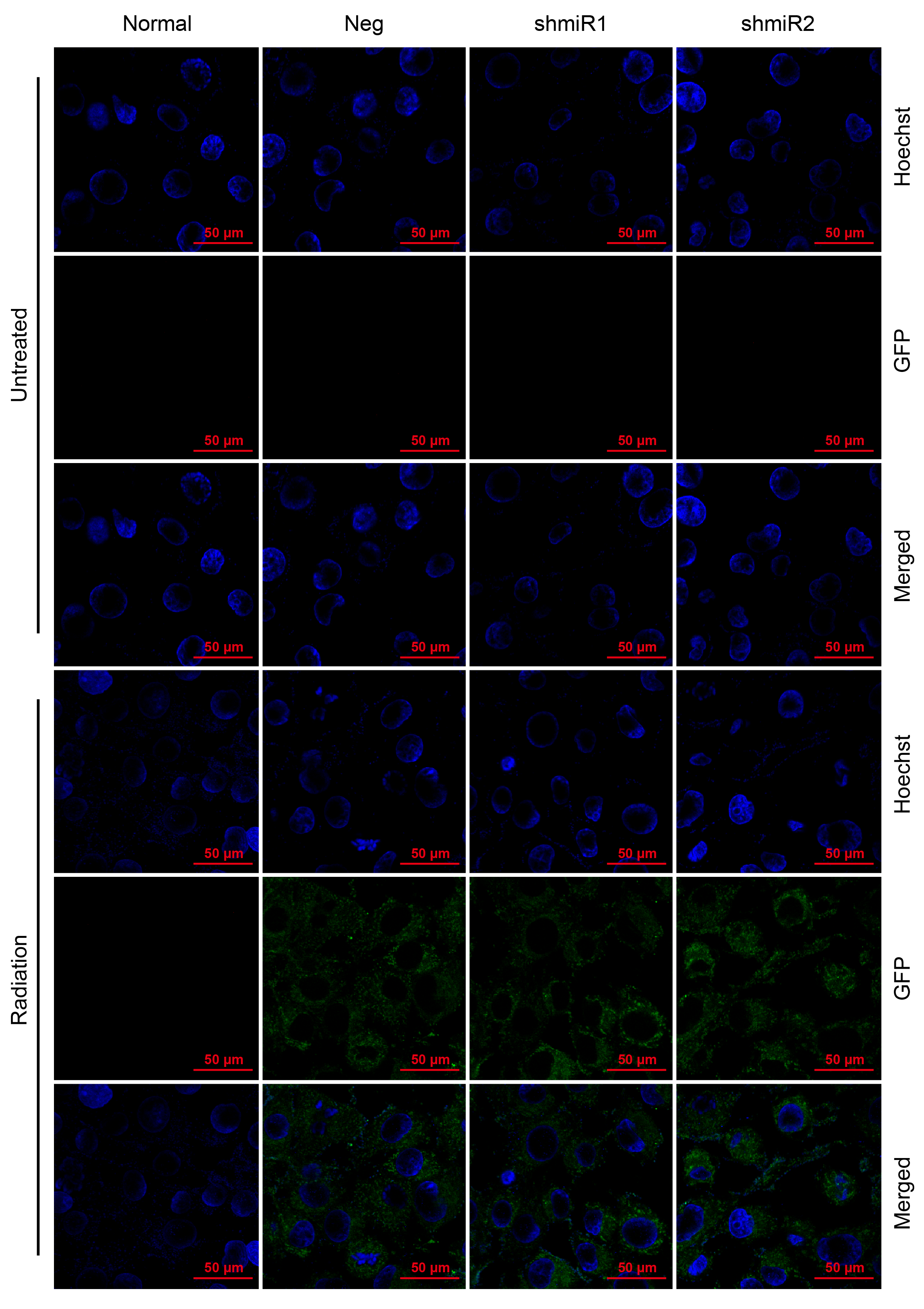

Supplement: S1 Fig — (TIF) [file pone.0163446.s001.tif]
