# Supplementary material for: MiRNA-Embedded ShRNAs for Radiation-Inducible LGMN Knockdown and the Antitumor Effects on Breast Cancer
Source: PLoS One. 2016 Sep 22;11(9):e0163446. doi: 10.1371/journal.pone.0163446 (PMC5033420; doi:10.1371/journal.pone.0163446)
Supplement: S1 File — Primers used in this study (Table A). GFP expression in SK-BR-3 cells treated for 24h by laser confocal microscopy (S1 Fig caption). Construction of shmiR plasmids with miR-155 flanking sequences (Protocol A). Construction of lentiviral plasmids containing radiation-inducible promoters and shRNA-miR-155 cassette (Protocol B). Lentivirus packaging and infection (Protocol C). (DOCX) [file pone.0163446.s002.docx]

**Table A. Primers used in this study**

| Primer | Sequence | Template/Cassette |
| --- | --- | --- |
| shRNA-1 F | 5’-TGCTGAAATATGACCCTTCTCAATACGTTTTGGCCACTGACTGACGTATTGAGGGGTCATATTT-3’ | shRNA1 |
| shRNA-1 R | 5’-CCTGAAATATGACCCCTCAATACGTCAGTCAGTGGCCAAAACGTATTGAGAAGGGTCATATTT-3’ |  |
| shRNA-2 F | 5’-TGCTGTTATACCAGCCATTTGAACCTGTTTTGGCCACTGACTGACAGGTTCAAGGCTGGTATAA-3’ | shRNA2 |
| shRNA-2 R | 5’-CCTGTTATACCAGCCTTGAACCTGTCAGTCAGTGGCCAAAACAGGTTCAAATGGCTGGTATAA-3’ |  |
| shRNA-miR F | 5’-GGCCTCGAGtaaCTGGAGGCTTGCTGAAG-3’ | shRNA-1-miR-155; shRNA-2-miR-155; shRNA-neg-miR-155 (XhoI; BamHI) |
| shRNA-miR R | 5’-GGCGGATCCGGCCATTTGTTCCATGTG-3’ |  |
| C_9_BC F | 5’-AGATCCAGTTTATCGATCCATATAAGGCCATATAAGG-3’ | C9BC-PUC19 |
| C_9_BC R | 5’-GCTCACCATGGTGGCATCTGAGTCCGGTAGCGC-3’ | In-Fusion |
| AcGFP1 F | 5’-GCCACCATGGTGAGCAAG-3’ | In-Fusion |
| AcGFP1 R | 5’-GAAGCTTGAGCTCGAGCTTGTACAGCTCATCCATG-3’ | pLVX-AcGFP1-C1 |
| LGMN F | 5’-GTGGCAGGTTCAAATGGCTG-3’ | cDNA of LGMN |
| LGMN R | 5’-CAGGAATCCCATTGCGGTGA-3’ |  |
| GADPH F | 5’-AGCCTCAAGATCATCAGCAA-3’ | cDNA of GAPDH |
| GADPH R | 5’-GTCATGAGTCCTTCCACGATAC-3’ |  |

**S1 Fig Caption. GFP expression in SK-BR-3 cells treated for 24h by laser confocal microscopy.** The figures showed above were merged photos in which cell nucleus was dyed with Hoechst 33342. The green fluorescence could be observe after 24 hours of radiation exposure in stably transfected cell lines. Normal: cells without treatment; Neg: cells stably expressed shRNA-neg-miR-155; shmiR1: cells stably expressed shRNA-1-miR-155; shmiR2: cells stably expressed shRNA-2-miR-155; Radiation: cells receiving 6 Gy of radiation treatment.

**Protocol A. Construction of shmiR plasmids with miR-155 flanking sequences**

shRNA sequences that had been designed and validated by our laboratory were used. The miR-155 flanking sequences in the plasmid pcDNA™6.2-GW/EmGFP-miR (Invitrogen, Carlsbad, CA, USA) were used, and two pairs of shRNA interference sequences were designed (BGI company, Shenzhan, China) according to the cloning strategy of this plasmid. The two pairs of primers were annealed, ligated into the linearized plasmid, and transformed into DH5α bacteria. The correct clones were selected. After positive clones were obtained, primers were designed to amplify the shRNA fragment containing the miR-155 sequences. The upstream PCR amplification primer was shown in **Table A**, which contains protective bases, an XhoI enzymatic digestion site, and a termination codon. The downstream PCR amplification primer contains protective bases and a bamhi enzymatic digestion site (**Table A**).

**Protocol B. Construction of lentiviral plasmids containing radiation-inducible promoters and shRNA-miR-155 cassette**

Whole gene synthesis of the chimeric promoter containing nine radiation-inducible elements and the CMV basic promoter (C_9_BC) was performed (BGI company) according to previous study results from our group and literature reports. In-Fusion seamless cloning technology (Takara, Dalian, China) was used to clone the promoter into the pLVX-AcGFP1-C1 vector to replace the original CMV promoter. The upstream and downstream primers for In-Fusion PCR of the C_9_BC promoter and the AcGFP1 fragment were shown in **Table A**. The ligated reaction products were transformed into Stb13 competent cells. Monoclonal amplified cultured bacteria were used for plasmid extraction. After validation, the pLVX-C_9_BC-AcGFP1-C1 plasmid containing the C_9_BC promoter was obtained. The constructed pLVX-C_9_BC-AcGFP1-C1 plasmid containing the radiation-inducible promoter and the fragment containing shRNA-miR-155 sequences were double-digested using the XhoI and BamHI enzymes. The fragments were separated in agarose gel, recovered, ligated using ligase (Takara) and transformed into Stb13 competent cells. The recombinant plasmids pLVX-C_9_BC-AcGFP1-shRNA-1-miR-155 (shmiR1), pLVX-C_9_BC-AcGFP1-shRNA-2-miR-155 (shmiR2), and pLVX-C_9_BC-AcGFP1-shRNA- neg-miR-155 (Neg) were obtained.

**Protocol C. Lentivirus packaging and infection**

The three-plasmid package system (Addgene, Cambridge, Massachusetts, USA. packaging plasmid psPAX2 and envelop plasmid pMD2.G) was used for virus package manipulation in a grade 2 biosafety cabinet. 293FT cells were used, and plasmid transfection was performed with liposomes. After 48 h and 72 h, virus supernatant was collected and ultra-centrifuged at 50000 x g for 2.5 h. After the virus pellet was dissolved in 100 μL of Opti-MEM, virus titers were determined using quantitative PCR. Target cells were inoculated, and the amount of virus was calculated based on the multiplicity of infection (MOI) value of the cell line. After 48 h of infection, stably transfected cell lines were screened using puromycin.
